# Supplementary material for: Mixed Wolbachia infections resolve rapidly during in vitro evolution
Source: bioRxiv. 2024 Mar 29:2024.03.27.586911. Preprint. [Version 1] doi: 10.1101/2024.03.27.586911 (PMC10996604; doi:10.1101/2024.03.27.586911)
Supplement: 1 [file NIHPP2024.03.27.586911v1-supplement-1.pdf]

bioRxiv preprint doi: https://doi.org/10.1101/2024.03.27.586911; this version posted March 29, 2024. The copyright holder for this preprint (which was not certified by peer review) is the author/funder, who has granted bioRxiv a license to display the preprint in perpetuity. It is made available under aCC-BY 4.0 International license.

Supporting Information

Fig S1. Schematic overview of steps required for successful horizontal transmission.
Host-switching of an endosymbiont requires successful horizontal transmission, intracellular proliferation, germline targeting for vertical transmission, and a mechanism for population establishment. Here, we use an *i n vitro Wolbachia*-infected cell culture system to study the early stages in this process (#1 and 2 in bold) that are often lost to chance. By focusing on closely related strains with promiscuous and stable host-associations, we can understand how cell identities, divergent hosts, and resident strains impact novel infection events.

Fig S2. Overview of *Drosophila in vivo* and *in vitro* resources.
The S2 and JW18 *D. melanogaster* cell lines were derived previously from fly embryos of unknown infection status and infected with wMel, respectively. The Dsim6B cell line was derived in this work, from embryos from the *D. simulans* white eye fly line infected with the Riv84 wRi strain (see methods panel through embryo homogenization). Uninfected cell lines were obtained by treatment with 10 μg/mL doxycycline (DOX) in the cell culture media for nine weeks, followed by at least two months recovery from antibiotic treatment mitochondrial effects. *Wolbachia* strains were swapped among cell lines with the shell vial technique (see methods panel through shell vial technique).

Fig S3. Natural and introduced *Wolbachia* infections in *D. melanogaster* cell lines are stable over time.
The wMel strain is consistently at ~10x higher titer than the wRi strain in *D. melanogaster* cells. Titers measured in 2021 were from cells maintained at 25-26°C, whereas titers measured in 2023 were from cells maintained at 23°C. Temperature has a similar impact on both strains titers, with both exhibiting proportionately lower titers at 23°C than 25-26°C.

Fig S4. Mixed Effects Regression analysis of relative strain frequency
To assess how cell line and initial infection ratios influenced wMel's competitive advantage over wRi, we utilized a linear mixed-effects model incorporating these variables as fixed effects. Prediction lines and 95% confidence intervals from the model and observed points for the two cell lines A) JW18 and B) S2 at starting ratios 1:100 (red) and 1:1000 (blue).

Fig S5. Recombinants detected between the wMel and wRi strains in D. melanogaster cell culture.
Recombinant alignments were detected by extracting the reads chimerically mapped to the wMel and wRi genomes in regions of high mappability (containing SNPs, indels, or structural variation).

Fig S6. Loss of the native wRi infection during Dsim[w-]6B cell line immortalization.

Fig S7. Shell-vial reinfection of Dsim6B[w-] cell line
*w*Mel (top) and *w*Ri (bottom) strains of *Wolbachia*.

Fig S8. Log-linear regression analysis of infected-uninfected mixtures
Log-linear regression analysis for A) wMel and B) wRi in 1:1 mixtures with uninfected cells. Regression summary statistics are annotated in Table S2.

Table S1. Selection coefficients estimated in competition experiments.

bioRxiv preprint doi: https://doi.org/10.1101/2024.03.27.586911; this version posted March 29, 2024. The copyright holder for this preprint (which was not certified by peer review) is the author/funder, who has granted bioRxiv a license to display the preprint in perpetuity. It is made available under aCC-BY 4.0 International license.

Table S2. Regression statistics from log-linear regression analysis of wMel:DOX and wRi:DOX experiments.

Table S3. Oligonucleotide sequences used for Tn5 based library preps.

Table S4. NCBI RefSeq genome accessions for reference genomes used in bioinformatics analyses.
